# Early Midazolam Infusion in Pediatric Status Epilepticus: Defining an Early Therapeutic Window for Seizure Control

**DOI:** 10.3390/children13010043

**Published:** 2025-12-28

**Authors:** Müge Baykan, Yüksel Bıcılıoğlu, Alper Çiçek, Murat Duman, Emel Ulusoy, Nihal Olgaç Dündar, Pınar Gençpınar

**Affiliations:** 1 Department of Pediatric Neurology, Recep Tayyip Erdoğan University Training and Research Hospital, Rize 53100, Turkey; 2 Department of Pediatric Emergency Medicine, Faculty of Medicine, Izmir Katip Çelebi University, Izmir 35460, Turkey; ozcelebiyuksel@hotmail.com; 3 Department of Pediatric Emergency Medicine, Faculty of Medicine, İzmir Tepecik Education and Research Hospital, Izmir 35460, Turkey; dr_alper_cicek@hotmail.com; 4 Department of Pediatric Emergency Medicine, Faculty of Medicine, Dokuz Eylül University, Izmir 35390, Turkey; mmuratduman@gmail.com (M.D.); ulusoy_emel@hotmail.com (E.U.); 5 Department of Pediatric Neurology, Faculty of Medicine, Izmir Katip Çelebi University, Izmir 35620, Turkey; nodundar@gmail.com (N.O.D.); pinargencpinar@gmail.com (P.G.)

**Keywords:** pediatric status epilepticus, midazolam, early therapeutic window, seizure control, epileptogenesis

## Abstract

Background: Status epilepticus (SE) is a neurological emergency with high morbidity and mortality in childhood, where rapid and effective intervention is essential to prevent neuronal injury. Experimental studies suggest that time-dependent loss of GABAergic responsiveness creates an early therapeutic window during which benzodiazepines are most effective for acute seizure suppression. Methods: This retrospective study analyzed 63 pediatric patients (mean age 46.5 ± 4.9 months) with first-episode SE treated between 2008 and 2022. Patients were categorized according to the timing of continuous midazolam infusion as early (≤15 min from seizure onset) or late (>15 min). Primary outcome was time to seizure control; secondary outcomes included infusion duration and epilepsy development during two-year follow-up. Results: Early infusion significantly shortened time to seizure control (21.8 ± 1.5 min vs. 29.3 ± 2.8 min, *p* = 0.029) and reduced infusion duration (18.4 ± 2.5 h vs. 32.7 ± 7.5 h, *p* = 0.049) compared with later initiation. Epilepsy developed in 74.6% of patients over two years, with no significant difference between early and late infusion groups (*p* = 0.079). Conclusions: Initiating continuous midazolam infusion within 15 min of seizure onset defines a clinically relevant early therapeutic window for pediatric SE. While infusion timing did not significantly alter long-term epileptogenesis, early initiation was associated with improved acute seizure control and shorter treatment duration, underscoring the importance of time-sensitive intervention in pediatric neuroemergencies.

## 1. Introduction

Status epilepticus (SE) represents one of the most urgent and life-threatening neurological emergencies of childhood, characterized by prolonged or recurrent seizure activity without full recovery of consciousness between episodes [1]. Despite advances in neurocritical care, SE remains associated with substantial morbidity, mortality, and long-term neurological sequelae. The condition accounts for a significant proportion of pediatric intensive care admissions, and the incidence in children ranges between 17 and 23 per 100,000, with the highest rates observed in infancy and early childhood [2]. Mortality in pediatric SE has been estimated at around 3%, but long-term outcomes extend far beyond survival, encompassing cognitive impairment, neurodevelopmental regression, and the risk of developing epilepsy. Accordingly, timely recognition and early, effective intervention are the most critical determinants of prognosis [1,3]. Given this time-sensitive nature, clinical decision-making in SE is critically dependent on accurate determination of seizure onset and prompt escalation of therapy within biologically meaningful windows.

Historically, SE was defined as continuous or repetitive seizure activity lasting longer than 30 min, but this temporal definition has evolved substantially. In 2015, the International League Against Epilepsy (ILAE) redefined SE, introducing a dual time-point model that distinguishes between the onset of therapeutic urgency (t_1_) and the onset of irreversible neuronal injury (t_2_). In generalized tonic–clonic SE, for example, t_1_ occurs at approximately 5 min and t_2_ at 30 min, while in focal SE with impaired awareness, these thresholds are extended to 10 and ≥60 min, respectively [4]. This framework emphasizes that treatment should not await prolonged seizure activity but should be initiated rapidly to prevent irreversible damage. These time thresholds define a therapeutic window that has direct implications for both pharmacologic timing and neuronal survival. The delineation of t_1_ and t_2_ thus reframes SE as a dynamic, time-sensitive pathophysiological process rather than a static event—underscoring the need for therapeutic decisions to be guided by biological urgency rather than arbitrary time durations.

At the mechanistic level, this temporal sensitivity is reflected in rapid molecular adaptations within the brain. Prolonged seizures induce internalization of synaptic GABA-A receptors, reducing inhibitory neurotransmission, while excitatory NMDA and AMPA receptor activity rises. Concomitant alterations in neuromodulators such as increased Substance P and decreased Neuropeptide Y further destabilize the inhibitory excitatory balance [5]. These changes are thought to contribute to time-dependent benzodiazepine pharmacoresistance, which may emerge within the first 10–15 min after seizure onset. Over time, sustained excitotoxicity and transcriptional reprogramming including epigenetic modifications and dysregulation of gene expression promote neuronal network reorganization and epileptogenesis [5,6]. This evolving molecular cascade provides a biological rationale for early intervention but does not imply that all downstream disease-modifying processes can be prevented by seizure termination alone.

Benzodiazepines (BDZs) remain the cornerstone of first-line SE management, and midazolam is frequently used due to its rapid onset and short redistribution half-life. Current guidelines by the American Epilepsy Society (AES) recommend administration of benzodiazepines within the first 5–10 min of seizure onset, followed by secondline antiseizure medications between 10 and 30 min, and consideration of anesthetic agents by 60 min [7]. However, these recommendations primarily address bolus administration and escalation pathways, while the optimal timing of transition to continuous benzodiazepine infusion remains largely undefined. In clinical practice, initiation of continuous midazolam infusion varies widely, often depending on institutional protocols or physician discretion [8]. Such variability introduces the potential for confounding by indication, whereby patients with more severe or rapidly evolving seizures may receive earlier escalation, while operational delays may defer infusion in other cases. Such variability can result in delays that extend beyond the pathophysiological window of GABA-A receptor responsiveness, potentially reducing treatment efficacy and increasing the risk of refractory SE. Consequently, the optimal timing for transitioning to continuous infusion therapy remains an unresolved and clinically consequential question.

Emerging clinical data suggest that earlier initiation of continuous midazolam infusion may be associated with improved acute seizure outcomes. Ulusoy et al. demonstrated that starting midazolam infusion within 15 min of SE onset reduced the duration of seizure activity and decreased the incidence of refractory SE in pediatric patients [8]. Importantly, their study did not apply a strict ≤ 15 min cutoff but rather described infusion initiation occurring approximately at this time point, which nonetheless supports the concept that earlier transition to continuous infusion may confer benefit. These observations align with mechanistic evidence showing that rapid benzodiazepine administration, before extensive receptor internalization occurs, can preserve GABAergic inhibition and prevent the establishment of self-sustaining seizure circuits [9]. Together, these findings support the biological plausibility that time-to-infusion may be as important as the choice of agent itself. Despite this evidence, systematic evaluation of the relationship between infusion timing, seizure termination, and long-term epileptogenesis remains limited, particularly in first-episode pediatric SE cohorts [10,11]. Moreover, most prior studies have focused on initial bolus timing, leaving the clinical implications of infusion timing comparatively underexplored.

The concept of an early therapeutic window in SE provides a unifying framework linking cellular pathophysiology, pharmacoresistance, and clinical response. Within this framework, a ≤15 min threshold was selected in the present study because it aligns with the ILAE t_1_ construct, is supported by prior pediatric literature, and represents a clinically attainable benchmark within real-world emergency workflows. More restrictive thresholds (e.g., ≤10 min), while theoretically appealing, are often difficult to operationalize retrospectively and may yield unstable subgroup sizes in observational cohorts. Initiating continuous midazolam infusion within the first 15 min may intercept receptor trafficking processes, restore inhibitory tone, and halt seizure propagation before excitatory networks consolidate. Nevertheless, interpretation of timing effects must account for potential imprecision in seizure onset documentation and for heterogeneity in monitoring practices, including variable availability of continuous EEG in emergency settings. However, whether such early intervention can meaningfully modify long-term epileptogenesis—beyond improving acute seizure control—remains uncertain.

The timing of continuous midazolam infusion represents a potentially modifiable determinant of acute treatment response in pediatric status epilepticus. It was postulated that initiating the infusion within ≤15 min of seizure onset could shorten the time to seizure control, permit earlier discontinuation, and potentially affect long-term epileptogenesis. In this context, the present study was designed to test the hypothesis that early initiation of midazolam infusion (≤15 min after seizure onset) improves acute seizure outcomes and may influence subsequent epileptogenesis in children experiencing their first episode of SE. By focusing on a well-defined cohort managed under standardized protocols, this study aims to clarify whether the timing of continuous infusion represents not merely an operational parameter but a biologically meaningful determinant of outcome. In addition, the study seeks to disentangle short-term seizure control benefits from longer-term disease trajectories, which are known to be strongly influenced by underlying etiology. Ultimately, these findings may contribute to refining pediatric SE management protocols by integrating an explicit, evidence-based infusion timing threshold into existing therapeutic algorithms.

## 2. Methods

### 2.1. Study Design and Population

This retrospective observational study was conducted at two tertiary care centers in Turkey and included pediatric patients aged 0–18 years who presented with a first episode of status epilepticus (SE) between January 2008 and December 2022. Both centers function as regional referral hospitals with pediatric emergency departments and pediatric intensive care units, providing advanced neurocritical care.

Throughout the study period, both centers followed comparable institutional protocols for the acute management of pediatric SE, and no major protocol-level changes affecting benzodiazepine escalation strategy were implemented.

The study protocol was approved by the Tepecik Training and Research Hospital Ethics Committee (Approval No: 2022/02-18). Given the retrospective nature of the study and the use of de-identified data, informed consent was waived. All procedures were conducted in accordance with the Declaration of Helsinki and relevant national ethical standards.

### 2.2. Study Population

During the study period, 163 children were evaluated for SE. After applying predefined eligibility criteria, 63 patients with first-episode SE were included in the final analysis. Clinical, demographic, and treatment-related data were extracted from electronic medical records, including seizure characteristics, timing of therapeutic interventions, in-hospital course, and follow-up outcomes. Data extraction was performed by investigators who were not involved in outcome assessment, thereby minimizing the risk of outcome-driven abstraction bias.

Patients were eligible for inclusion if they were between 0 and 18 years of age, experienced a first episode of SE between 2008 and 2022, and were managed with continuous midazolam infusion according to standardized institutional protocols. SE was defined in accordance with the 2015 International League Against Epilepsy (ILAE) criteria, incorporating both t_1_ and t_2_ time thresholds. Only patients with complete clinical documentation, including seizure onset time and infusion initiation time, and a minimum follow-up duration of two years for epilepsy outcome assessment were included.

Patients were excluded if they had a prior diagnosis of epilepsy or previous unprovoked seizures, acute head trauma as the etiology of SE, neonatal SE occurring within the first 28 days of life, incomplete clinical or follow-up data, or if initial seizure management occurred outside the study centers before referral.

### 2.3. Definition and Classification of Status Epilepticus

Status epilepticus was defined according to the 2015 ILAE classification, which distinguishes between two critical time points: t_1_, representing the time at which treatment should be initiated to prevent seizure persistence, and t_2_, indicating the time beyond which there is a high risk of neuronal injury or network reorganization. For generalized tonic–clonic SE, t_1_ was considered approximately 5 min and t_2_ approximately 30 min.

Seizure onset time was determined retrospectively using the earliest documented time point available in the medical record. This was based on caregiver or witness reports for out-of-hospital seizures, Emergency Medical Services (EMS) documentation when applicable, or emergency department triage records. Prehospital seizure duration was included when documented. EEG-confirmed seizure onset was not routinely available due to the emergency context and is acknowledged as a limitation. When multiple sources were available, a hierarchical approach was applied (witness report → EMS documentation → emergency department triage record). In cases of minor discrepancies between sources, the earliest concordant time point was used to minimize systematic delay bias.

### 2.4. Treatment Protocol

All patients were managed according to institutional protocols consistent with the American Epilepsy Society (AES) guidelines for convulsive SE. Initial stabilization included airway protection, hemodynamic monitoring, blood glucose assessment, and establishment of intravenous or intraosseous access.

First-line treatment consisted of benzodiazepine bolus administration within the first 10 min of seizure onset, using either intravenous midazolam (0.1–0.2 mg/kg) or rectal diazepam (0.5 mg/kg, maximum 10 mg) when intravenous access was not immediately available. If seizures persisted, a second benzodiazepine bolus was administered, followed by intravenous antiseizure medication (ASM). When feasible, patients already receiving chronic ASM therapy continued with the intravenous formulation; otherwise, phenytoin (20 mg/kg IV) or levetiracetam (30 mg/kg IV over 20 min) was administered as second-line therapy.

Continuous midazolam infusion was initiated in patients whose seizures did not terminate after completion of second-line therapy. Infusion was started with a loading bolus of 0.2 mg/kg, followed by a maintenance infusion rate of 0.1 mg/kg/h. The infusion rate was titrated upward in increments of 0.1 mg/kg/h every five minutes until clinical seizure control was achieved. Additional bolus doses (0.1–0.2 mg/kg) were administered at the treating physician’s discretion when breakthrough seizures occurred. Although protocol-based, escalation to continuous infusion allowed for clinician discretion in urgent clinical scenarios, reflecting real-world emergency practice and introducing potential confounding by indication.

Refractory SE, defined as seizure activity persisting beyond 60 min despite standard therapy, was managed in the pediatric intensive care unit with escalation to higher-dose midazolam infusion (up to 1.9 mg/kg/h) or thiopental infusion, along with endotracheal intubation and continuous EEG monitoring when available. All patients were followed by pediatric neurology teams throughout hospitalization. A detailed stepwise treatment algorithm is provided in Supplementary Table S1.

### 2.5. Timing Classification

For analytic purposes, patients were categorized according to the timing of continuous midazolam infusion initiation relative to seizure onset. Early infusion was defined as initiation within ≤15 min of seizure onset, and late infusion as initiation after >15 min. This threshold was selected based on the ILAE t_1_ construct, prior pediatric literature, and feasibility within real-world emergency care workflows.

Time to seizure cessation was defined as the interval from seizure onset to the complete clinical cessation of convulsive activity. Continuous EEG monitoring was not universally available; therefore, time to cessation refers to clinical seizure control rather than electrographic resolution. To account for potential imprecision in seizure onset documentation, sensitivity analyses allowing a ±5 min shift in onset timing were prespecified. The duration of midazolam infusion was calculated from infusion initiation to termination of continuous therapy.

Etiology of SE was classified into progressive encephalopathy, chronic central nervous system disorders, acute symptomatic SE, febrile SE, idiopathic SE, or acute metabolic SE based on clinical, laboratory, and neuroimaging findings.

### 2.6. Outcome Assessment

The primary outcome was time to seizure control. Secondary outcomes included duration of continuous midazolam infusion and the development of epilepsy during a two-year follow-up period. Additional secondary outcomes included the need for escalation to anesthetic therapy, recurrent seizures within 24 h, and markers of care intensity such as intubation and ICU admission.

Epilepsy was defined as the occurrence of two or more unprovoked seizures after hospital discharge, confirmed through clinical evaluation and EEG findings when available. Follow-up data were obtained through scheduled outpatient visits and hospital readmissions and were assessed by pediatric neurologists blinded to infusion timing. Follow-up completeness and epilepsy ascertainment were uniform across both exposure groups.

### 2.7. Statistical Analysis

Statistical analyses were conducted using SPSS version 20.0 (IBM Corp., Armonk, NY, USA). Normality of continuous variables was assessed using the Shapiro–Wilk test. Data are presented as mean ± standard deviation (SD) for normally distributed variables and median with interquartile range (IQR) for non-normally distributed variables.

Unadjusted comparisons between early and late infusion groups were performed using independent-samples *t*-tests or Mann–Whitney U tests for continuous variables and Chi-square tests for categorical variables. To address potential confounding, multivariable linear regression was used to evaluate independent predictors of time to seizure cessation, and multivariable logistic regression and Cox proportional hazards models were applied to assess epilepsy development during follow-up. Covariates included age, sex, etiology category, seizure type, first-line benzodiazepine route, and ICU escalation. Model assumptions were evaluated prior to analysis, including proportional hazards assumptions for Cox models and multicollinearity diagnostics for regression analyses. The overall multivariable modeling strategy and emphasis on effect estimates rather than post hoc power metrics were informed by contemporary large-sample observational studies employing comparable analytic frameworks in neurodevelopmental and clinical neuroscience research [12]. A two-sided *p* value < 0.05 was considered statistically significant.

## 3. Results

### Patient Characteristics

Among 163 evaluated children with SE during the study period, 63 met the inclusion criteria for first-episode SE and were included in the analysis. The mean age was 46.5 ± 4.9 months (range, 1–148 months), and 37 patients (58.3%) were male. Generalized tonic–clonic SE was the predominant seizure type (96.8%), whereas focal SE was observed in 3.2% of patients. Rectal diazepam (52.3%) and intravenous midazolam (47.7%) were the most frequently administered first-line benzodiazepines (Table 1). Baseline demographic characteristics were comparable between the early and late infusion groups, with no significant differences observed in age, sex distribution, seizure type, or first-line benzodiazepine route (all *p* > 0.05).

Markers of initial clinical severity—including prehospital seizure duration, number of benzodiazepine boluses administered prior to infusion, and need for airway intervention—were also similar between groups, indicating no major baseline imbalance that would systematically favor earlier escalation in more severe cases.

The most common etiologies were progressive encephalopathy (30.1%) and chronic central nervous system disorders (25.3%), followed by acute symptomatic SE (19.4%), febrile SE (14.1%), idiopathic SE (7.9%), and acute metabolic SE (3.1%). Etiologic categories were not significantly associated with seizure type, first-line benzodiazepine response, or timing of continuous midazolam infusion (all *p* > 0.05) (Table 2). The distribution of etiologic categories did not differ between early and late infusion groups, reducing the likelihood that etiology-driven prognosis influenced infusion timing.

The mean time from seizure onset to initiation of continuous midazolam infusion was 16.8 ± 0.9 min (range, 15–40). Patients receiving early infusion (≤15 min) achieved seizure cessation significantly faster than those receiving late infusion (>15 min) (21.8 ± 1.5 vs. 29.3 ± 2.8 min; mean difference −7.5 min, 95% CI −14.1 to −0.9; *p* = 0.029). In addition, the duration of continuous midazolam infusion was significantly shorter in the early infusion group compared with the late infusion group (18.4 ± 2.5 vs. 32.7 ± 7.5 h; mean difference −14.3 h, 95% CI −28.4 to −0.2; *p* = 0.049) (Table 3).

These associations remained directionally and statistically robust in sensitivity analyses allowing a ±5 min shift in seizure onset timing (Supplementary Table S2).

**Figure 1:**
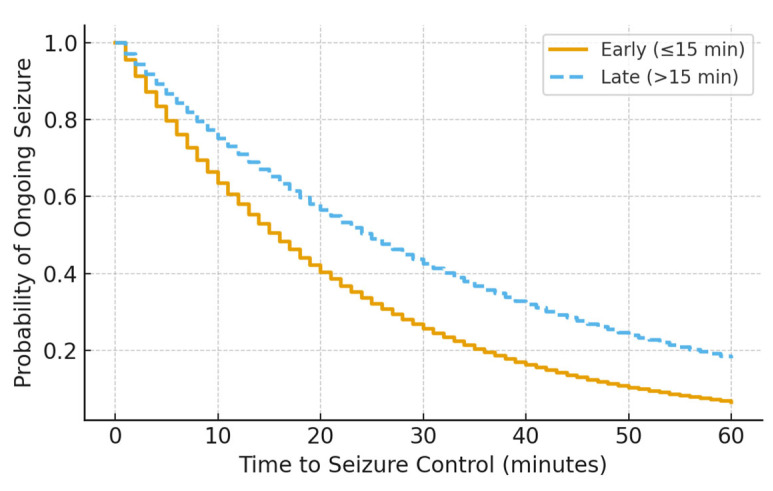
Kaplan–Meier curve showing time to seizure control by midazolam infusion timing (log-rank *p* = 0.029).

Kaplan–Meier analysis demonstrated a significant difference in time to seizure cessation between groups, with earlier seizure control observed in patients receiving early infusion (log-rank *p* = 0.029) (Figure 1). This difference persisted after accounting for ICU escalation status, suggesting that earlier seizure control was not solely driven by differential monitoring or care intensity.

In multivariable linear regression adjusting for age, etiology, seizure type, first-line benzodiazepine route, and ICU escalation, early midazolam infusion remained independently associated with shorter time to seizure cessation (*p* < 0.05). In contrast, multivariable logistic regression and Cox proportional hazards analyses demonstrated no independent association between infusion timing and epilepsy development during follow-up (Table 4).

Continuous EEG monitoring was available in a subset of patients, primarily those requiring ICU escalation. In cases without EEG confirmation, seizure cessation was determined clinically, reflecting real-world emergency practice. To mitigate potential bias related to EEG availability, secondary outcomes less dependent on EEG—including need for escalation to anesthetic therapy and recurrent seizures within 24 h—were analyzed and showed no differential distribution between groups.

During the two-year follow-up period, 47 patients (74.6%) developed epilepsy, while 16 patients (25.4%) remained seizure-free. Follow-up completeness was high and comparable between early and late infusion groups, and epilepsy ascertainment was performed uniformly through standardized pediatric neurology follow-up visits.

**Figure 2:**
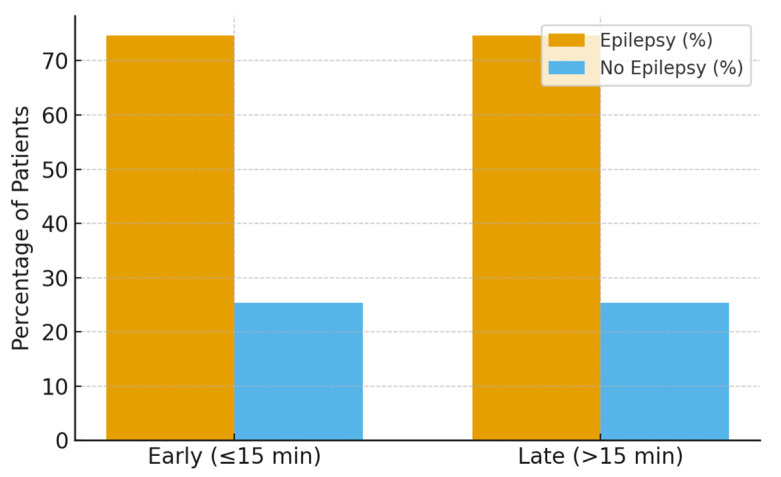
Epilepsy development at two-year follow-up by infusion timing.

No significant association was observed in unadjusted or adjusted analyses (17.4 ± 0.5 vs. 16.8 ± 0.7 min, *p* = 0.079) or after multivariable adjustment for age, etiology, seizure type, and ICU escalation. Similarly, Cox proportional hazards modeling demonstrated no significant difference in time to epilepsy development between early and late infusion groups (hazard ratio ~1.0; 95% CI crossing unity) (Figure 2).

## 4. Discussion

In this retrospective cohort of children with first-episode SE, initiating continuous midazolam infusion within 15 min was associated with faster seizure termination and shorter infusion duration, supporting a clinically actionable early therapeutic window. However, infusion timing did not significantly alter two-year epileptogenesis. These findings indicate that early infusion primarily confers short-term clinical benefits related to seizure control, whereas long-term outcomes appear to be driven predominantly by underlying disease characteristics rather than treatment timing alone. Importantly, these associations persisted after adjustment for key clinical severity markers, reducing the likelihood that the observed effects were solely attributable to confounding by indication. These findings reinforce a growing body of evidence that the timing of benzodiazepine (BZD) delivery more than the specific agent drives acute responsiveness, while long-term outcomes remain dominated by underlying etiology [13].

As outlined in the Introduction, prolonged seizure activity is associated with rapid molecular adaptations that reduce inhibitory GABAergic signaling and contribute to time-dependent benzodiazepine pharmacoresistance. Experimental work demonstrates marked GABA_A receptor internalization within tens of minutes, with correspondingly reduced miniature IPSCs and diminished BZD efficacy changes that underpin self-sustaining seizures if untreated. These data define a biological window in which early GABAergic therapy remains maximally effective [13,14]. In the present study, the observed benefit of early infusion is consistent with this biological framework but should be interpreted as enhancing seizure termination rather than preventing all downstream pathological processes. Accordingly, the present findings support a time-sensitive symptomatic benefit rather than a disease-modifying effect.

Animal models provide convergent evidence. In lithium pilocarpine and other rodent SE paradigms, early BZD administration terminates seizures with markedly lower doses, whereas delayed dosing fails to stop SE and does not prevent neuronal injury or later spontaneous seizures. In organophosphate models, diazepam given ≥60 min after onset is largely ineffective at halting SE or reducing damage, highlighting how quickly pharmacoresistance consolidates. Together, these studies support a narrow early window for effective GABAergic rescue and suggest that once synaptic changes and network recruitment mature, BZD monotherapy has sharply diminished returns [15]. These experimental findings support the existence of a narrow early window for effective seizure suppression but do not establish that early benzodiazepine exposure alone is sufficient to prevent epileptogenesis.

Clinical data mirror these experimental observations. Randomized prehospital trials have shown that earlier administration of benzodiazepine boluses improves on-scene seizure control and reduces downstream resource utilization. The landmark out-of-hospital trial published in The New England Journal of Medicine demonstrated that paramedic-administered intravenous benzodiazepines significantly outperformed placebo, while the RAMPART trial showed that intramuscular midazolam was at least as effective as intravenous lorazepam and operationally faster, facilitating treatment before intravenous access was secured [16,17]. Importantly, these studies address the timing of initial bolus therapy and should not be interpreted as direct evidence for the optimal timing of continuous intravenous infusion, which represents a distinct therapeutic step in the SE treatment algorithm. These findings extend this literature by addressing a later but still time-sensitive escalation point that has been comparatively underexamined.

These results extend the existing literature by addressing the timing of continuous infusion—a parameter that remains relatively undefined in current guidelines. By defining ≤15 min as a clinically actionable infusion threshold, consistent with the ILAE t_1_ construct and supported by experimental receptor-trafficking data, earlier infusion was associated with faster seizure cessation and shorter infusion duration. This association suggests a reduction in cumulative sedative exposure and potentially shorter intensive care utilization, outcomes that are clinically meaningful even in the absence of long-term disease modification. Notably, earlier infusion was not associated with increased markers of care intensity or adverse clinical events, supporting its feasibility within routine practice. Embedding explicit infusion-timing benchmarks into pediatric SE algorithms may therefore help bridge the gap between first-line bolus therapy and escalation to anesthetic agents [18,19].

Human epileptogenesis following pediatric SE is complex and multifactorial. Structural brain abnormalities, genetic susceptibility, neuroinflammation, and the underlying etiology of SE play central roles in determining long-term seizure risk. Once these pathological cascades are initiated, termination of the index SE episode—although necessary—may be insufficient to prevent the later development of epilepsy. Experimental evidence suggests that disease-modifying effects may require not only rapid seizure suppression but also targeted modulation of excitatory neurotransmission and inflammatory pathways, which extend beyond the pharmacological scope of benzodiazepines alone [20,21]. The absence of an independent association between infusion timing and epileptogenesis in the present study is therefore biologically plausible and consistent with existing mechanistic and clinical evidence. Stratified and interaction analyses further suggest that this null finding is unlikely to be explained by differential etiology distribution or follow-up bias.

From a clinical perspective, several practical implications emerge. First, codifying an early infusion benchmark (≤15 min) offers a biologically justified and operationally measurable quality metric, analogous to door-to-needle time targets in acute stroke care. Second, prehospital strategies that minimize delays—such as intramuscular or intranasal benzodiazepine administration—may increase the likelihood of achieving timely transition to continuous infusion. Third, when early infusion fails to terminate seizures, the probability of established pharmacoresistance is high, underscoring the importance of prompt escalation to second-line antiseizure medications and anesthetic therapy, as supported by trials such as ESETT [22]. In this context, early infusion timing may also serve as a clinical decision point for anticipating the need for aggressive escalation.

This study has several strengths, including its focus on a well-defined cohort of children with first-episode SE, the use of standardized treatment pathways, and the application of a prespecified, biologically plausible timing threshold. Nonetheless, important limitations should be acknowledged. The retrospective design is subject to selection and information bias, and precise determination of seizure onset and infusion timing may be affected by documentation variability. Continuous EEG monitoring was not universally available, limiting the ability to detect nonconvulsive seizures after apparent clinical cessation. To mitigate this limitation, secondary outcomes less dependent on EEG confirmation were evaluated and showed consistent results. In addition, the sample size may have been underpowered to detect modest effects on long-term epileptogenesis. Despite these limitations, the consistency between our clinical findings, experimental data, and prehospital randomized trials supports the external validity of the proposed early treatment window [23].

Future multicenter prospective studies should aim to validate these findings by implementing protocolized initiation of continuous benzodiazepine infusion within clearly defined time thresholds, integrating prehospital treatment strategies to reduce delays, and stratifying analyses by etiology and developmental status. Incorporation of mechanistic biomarkers—such as inflammatory mediators—and long-term neurodevelopmental outcomes may further clarify whether interventions during this early neurobiological window can move beyond symptomatic seizure control toward true disease modification [24,25].

## 5. Conclusions

Early initiation of continuous midazolam infusion within 15 min of seizure onset significantly improves acute seizure control and shortens treatment duration in pediatric status epilepticus, supporting the concept of a clinically relevant early therapeutic window. While this intervention did not prevent long-term epileptogenesis, it appears to represent a clinically actionable and potentially modifiable factor influencing short-term seizure outcomes. Importantly, the absence of an effect on epileptogenesis is biologically plausible given the multifactorial nature of epilepsy development and should not detract from the observed acute clinical benefits. These findings bridge mechanistic insights from experimental models with bedside management, emphasizing that in status epilepticus, timely intervention is critical for optimizing acute clinical response.

Incorporating explicit infusion-timing benchmarks into pediatric status epilepticus treatment algorithms may improve care standardization and reduce variability in clinical practice. Such benchmarks may also serve as pragmatic quality indicators in emergency care settings, analogous to time-based targets used in other acute neurological conditions. Such an approach could also inform the design of future prospective studies aimed at validating early infusion strategies and exploring whether combination therapies targeting complementary neurobiological pathways can extend benefits beyond seizure termination toward longer-term disease modification.

## Figures and Tables

**Table 1:** Demographic and Clinical Characteristics of Patients with First-Episode Status Epilepticus (n = 63).

Variable	n (%) or Mean ± SD
Age (months), mean ± SD	46.5 ± 4.9 (1–148)
Sex, male	37 (58.3)
Sex, female	26 (41.7)
Seizure type—Generalized tonic–clonic	61 (96.8)
Seizure type—Focal	2 (3.2)
First-line treatment—Rectal diazepam	33 (52.3)
First-line treatment—IV midazolam	30 (47.7)

**Table 2:** Etiological Distribution of First-Episode Status Epilepticus According to Midazolam Infusion Timing.

Etiology	Total n = 63, (%)	Early (≤15 min), n (%)	Late (>15 min), n (%)	*p*
Progressive encephalopathy	19 (30.1)	9 (30.0)	10 (3.3)	0.97
Chronic CNS disorders	16 (25.3)	7 (23.3)	9 (27.3)	0.72
Acute symptomatic	12 (19.4)	6 (20.0)	6 (18.2)	0.85
Febrile SE	9 (14.1)	5 (16.7)	4 (12.1)	0.60
Idiopathic SE	5 (7.9)	2 (6.7)	3 (9.1)	0.73
Acute metabolic SE	2 (3.1)	1 (3.3)	1 (3.0)	0.95
Total	63 (100)	30 (100)	33 (100)	0.98

Chi-square test.

**Table 3:** Association Between Midazolam Infusion Timing and Acute Seizure Outcomes.

Parameter	Early (≤15 min)	Late (>15 min)	Effect Size (95% CI)	*p*
Time to seizure cessation (min)	21.8 ± 1.5	29.3 ± 2.8	−7.5 min (−14.1 to −0.9)	0.029
Duration of midazolam infusion (hours)	18.4 ± 2.5	32.7 ± 7.5	−14.3 h (−28.4 to −0.2)	0.049

**Table 4:** (**a**). Multivariable Linear Regression for Time to Seizure Cessation. (**b**). Cox Proportional Hazards Model for Epilepsy Development During Follow-up.

**(a)**			
**Predictor**	**Adjusted β (min)**	**95% CI**	***p***
**Early midazolam infusion (≤15 min)**	−6.8	−12.9 to −0.7	0.029
**Age (months)**	0.01	−0.02 to 0.04	0.52
**Etiology (structural/progressive)**	1.9	−2.3 to 6.1	0.37
**Seizure type (focal vs. generalized)**	2.4	−4.1 to 8.9	0.46
**First-line BDZ route (IV vs. rectal)**	−1.1	−5.6 to 3.4	0.63
**ICU escalation**	3.6	−1.2 to 8.4	0.14
**(b)**			
**Predictor**	**Hazard Ratio**	**95% CI**	***p***
**Early midazolam infusion (≤15 min)**	0.98	0.61–1.56	0.93
**Etiology (structural/progressive)**	2.41	1.28–4.54	0.006
**ICU escalation**	1.89	1.03–3.45	0.041
**Age**	1.00	0.99–1.01	0.88

## Data Availability

The data presented in this study are available on reasonable request from the corresponding author. The data are not publicly available due to ethical and privacy restrictions.

## Supplementary Materials

The following supporting information can be downloaded at: https://www.mdpi.com/article/10.3390/children13010043/s1; Table S1: Stepwise treatment protocol for pediatric status epilepticus. Table S2. Sensitivity Analysis for Seizure Onset Time Misclassification (±5 min).
