# Supplementary material for: Early Midazolam Infusion in Pediatric Status Epilepticus: Defining an Early Therapeutic Window for Seizure Control
Source: Children (Basel). 2025 Dec 28;13(1):43. doi: 10.3390/children13010043 (PMC12840102; doi:10.3390/children13010043)
Supplement: Supplementary file 1 [file children-13-00043-s001.zip › children-4012905-supplementary.pdf]

Supplementary Table S1. Stepwise treatment protocol for pediatric status epilepticus

Time from seizure onset	Treatment step	Medication and dose	Notes / Actions
**0–5 min**	Initial stabilization	Airway, breathing, circulation assessment; monitor vital signs; obtain IV/IO access; check glucose	If glucose <60 mg/dL → 10% dextrose, 2 mL/kg IV bolus
**0–10 min**	First-line therapy	**Midazolam** 0.1–0.2 mg/kg IV bolus (max 10 mg); if no IV access → **Midazolam** 0.2 mg/kg IM or **Diazepam** 0.5 mg/kg rectal (max 10 mg)	Repeat once after 5 min if seizure persists
**10–15 min**	Second-line therapy	Repeat benzodiazepine as above, then give one IV antiseizure medication: • **Phenytoin** 20 mg/kg IV (max rate 1 mg/kg/min or 50 mg/min) • or **Levetiracetam** 30 mg/kg IV over 20 min	Choose based on prior ASM exposure and IV formulation availability
**≤15 min (Early**	Continuous infusion initiation	**Midazolam** 0.2 mg/kg IV bolus → start infusion at 0.1 mg/kg/h;	Additional 0.1–0.2 mg/kg boluses

**infusion target)**		titrate by 0.1 mg/kg/h every 5 min until seizure control	permitted during titration
**15–60 min**	Refractory phase management	Continue midazolam titration (max 1.9 mg/kg/h); add second IV ASM after first-line complete	Consider pyridoxine 100 mg IV if <2 years and cryptogenic SE
**>60 min**	Established/refractory SE	Transfer to ICU; airway protection and intubation as needed; continuous EEG monitoring; consider **Thiopental** 3 mg/kg bolus → 1–5 mg/kg/h infusion	Maintain seizure suppression for ≥24 h before gradual taper

Abbreviations: IV, intravenous; IM, intramuscular; IO, intraosseous; ASM, antiseizure medication; SE, status epilepticus; ICU, intensive care unit.

Supplementary Table S2. Sensitivity Analysis for Seizure Onset Time Misclassification (±5 minutes)

Scenario	Early Infusion (≤15 min)	Late Infusion (>15 min)	Mean Difference in Time to Seizure Cessation (min)	95% CI	p
**Primary analysis (observed timing)**	21.8 ± 1.5	29.3 ± 2.8	−7.5	−14.1 to −0.9	0.029
**Onset shifted −5 min**	22.1 ± 1.6	29.0 ± 2.7	−6.9	−13.4 to −0.8	0.034
**Onset shifted +5 min**	21.5 ± 1.7	29.6 ± 2.9	−8.1	−14.9 to −1.0	0.027

To evaluate the robustness of the primary findings against potential imprecision in seizure onset documentation, sensitivity analyses were performed by shifting seizure onset times by ±5 minutes. Across all scenarios, early initiation of continuous midazolam infusion remained significantly associated with shorter time to seizure cessation, indicating that the primary results were not driven by plausible timing misclassification.
